# MYC in Brain Development and Cancer

**DOI:** 10.3390/ijms21207742

**Published:** 2020-10-20

**Authors:** Olga Zaytseva, Nan-hee Kim, Leonie M. Quinn

**Affiliations:** Department of Cancer Biology and Therapeutics, The John Curtin School of Medical Research, The Australian National University, Canberra, ACT 2600, Australia; nan-hee.kim@anu.edu.au (N.-h.K.); leonie.quinn@anu.edu.au (L.M.Q.)

**Keywords:** MYC, brain development, neural stem cells, brain cancer

## Abstract

The MYC family of transcriptional regulators play significant roles in animal development, including the renewal and maintenance of stem cells. Not surprisingly, given MYC’s capacity to promote programs of proliferative cell growth, MYC is frequently upregulated in cancer. Although members of the MYC family are upregulated in nervous system tumours, the mechanisms of how elevated MYC promotes stem cell-driven brain cancers is unknown. If we are to determine how increased MYC might contribute to brain cancer progression, we will require a more complete understanding of MYC’s roles during normal brain development. Here, we evaluate evidence for MYC family functions in neural stem cell fate and brain development, with a view to better understand mechanisms of MYC-driven neural malignancies.

## 1. Introduction

The MYC family of basic helix-loop-helix (bHLH) transcription factors, comprised of MYC, MYCN and MYCL, are central to cell fate decisions, controlling transcriptional networks that govern cell growth, division, differentiation and death (reviewed in [1,2,3,4,5]). The expression of *MYC* family genes is tightly regulated during development in order to ensure high expression in proliferative stem and progenitor cells and downregulation in differentiated daughters [6]. In accordance with the immutable link between elevated MYC and proliferative potential, MYC is one of the four Yamanaka factors required to reprogram differentiated fibroblasts into pluripotent stem cells [7,8]. Moreover, the observation that MYC family transcription factors are conserved in primordial stem cells, including the demosponge *Ephydatia fluviatilis*, the hydrozoan cnidarian *Hydra vulgaris* and several flatworm species [9], suggests ancestral roles for MYC proteins in stem cell identity. The three mammalian MYC family members are also highly conserved as a single Myc ortholog in the Diptera insect *Drosophila melanogaster*. Myc controls stem cell identity in *Drosophila* imaginal tissues (reviewed in [10]), and functional conservation with mammalian MYC is evident from the genetic rescue of *Myc*-null mutant flies with human MYC, together with the capacity for fly Myc to drive the transformation of mouse fibroblasts [11,12].

MYC has long been suspected to widely impact transcription [13,14]. Although originally predicted to regulate 10–15% of the coding genes in the genome based on Dam-methylase binding studies [15,16], genome-wide Chromatin Immunoprecipitation (ChIP)-sequencing studies demonstrated the broader reach of MYC control. MYC modulates extensive transcriptional programs by interacting with RNA Polymerase II-enriched active promoter/enhancer regions; thus, MYC can drive cells towards predetermined cellular states by amplifying established cell-specific transcriptional programs [17,18,19,20,21]. Transcriptional outcomes are dictated by the composition of heterodimeric MYC complexes; for example, interaction with a second bHLH protein MAX activates transcription, while interaction with the BTB/POZ-domain zinc finger protein MIZ-1 can repress the promoter activity [22]. Moreover, gene expression amplified by MYC-MAX can be modulated by the MAX dimerization protein 1 (MAD1, encoded by the human MXD1 gene), which interacts with MAX to transcriptionally repress target genes [23]. In addition to the broad capacity to increase transcription, a nuanced control of certain MYC target genes is achieved through differing the promoter affinity [24].

As a result, MYC’s capacity to regulate broad transcriptional networks to control developmental and homeostatic processes is inextricably linked with the cellular context [25]. In the context of stem cells, we predict that MYC’s capacity to amplify programs that promote proliferative growth and repress differentiation underlies its key role as a Yamanaka factor. Thus, MYC has been broadly implicated in maintaining asymmetric division, to ensure the neural stem cell renewal that is required to furnish the developing brain with diverse neural and glial cell populations. Not surprisingly, given the capacity to increase growth and proliferation, MYC is frequently upregulated in primary brain tumours (reviewed elsewhere, [26,27,28]). Understanding how MYC family members regulate the transcription of networks central to neural stem cell fate decisions during development will be essential in order to gain insights into the mechanisms of MYC-dependent brain tumour initiation and progression.

## 2. Distinct Expression of the MYC Family during Mammalian Brain Development

During embryogenesis, the mammalian central nervous system (CNS) emerges following the folding of the neural plate and neural tube formation. The neural crest cells delaminate and migrate throughout the embryo body, forming diverse lineages, including the peripheral nervous system (PNS), endocrine cells and facial structures. Neural tube vesicles form presumptive structures of the CNS, including cerebral hemispheres, the midbrain and the hindbrain, while the fluid-filled cavity of the neural tube forms ventricles within the developing brain. Proliferative neural precursor cells derived from the neural tube are maintained in the ventricular and subventricular zones of the embryonic brain [29]. During weeks 5–6 of gestation in humans, corresponding to mouse embryonic day (E)10.5, these cells give rise to radial glia progenitors, which undergo asymmetric cell division prior to differentiation into the neurons and glia that populate the neonatal cortex [30,31].

**Figure 1:**
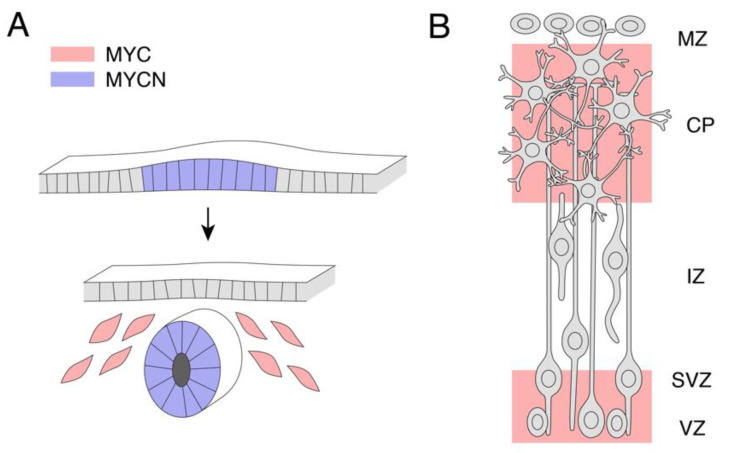
MYC and MYCN are highly expressed in neural precursors during mammalian development. (**A**) MYCN expression is high in the neuroectoderm and the neural tube. During the neural tube formation, MYC expression is high in the neural crest. (**B**) At later stages of development, the cortex is layered into distinct zones. VZ, ventricular zone, SVZ, subventricular zone, IZ, intermediate zone, CP, cortical plate, MZ, medullary zone. MYC protein expression was observed in VZ, SVZ and CP.

At the early stages of murine development (E8.5), *N-Myc* mRNA is highly expressed in the neuroectoderm [32]. MYCN protein expression remains high in the cerebellar and cerebral progenitor cells throughout E12.5 [33]. During this stage, *L-Myc* mRNA is also relatively highly expressed, particularly in the ventricular zones and the olfactory epithelium [34]. By E18.5, MYC expression is observed in the ventricular and subventricular zones [35]. Interestingly, MYC protein is also detected in the cortical plate (site of mature neurons), although the identity of the cell types with the greatest MYC expression is unknown [35] (Figure 1A,B). In line with the expression in the developing brain, a recent single-cell RNA-sequencing analysis of primary glioblastoma tumours identified high *MYC* mRNA across several lineages, including radial glia, progenitor and oligodendrocyte precursor cells [36], suggesting a requirement to drive proliferation across multiple neural cell types.

## 3. MYC and MYCN Regulate Brain Development in Mammals

*Myc*-null mice exhibit embryonic lethality prior to E10.5 and a wide range of developmental defects, including failure of neural tube closure [37]. The lethality has, however, been attributed to MYC functions in the extra-embryonic development, as defects in embryos with the epiblast-specific deletion of *Myc* are restricted to the haematopoietic lineage, i.e., brain organogenesis occurs normally [38]. In line with the high expression in the brain, *N-Myc*-null mice display defects in the central and peripheral nervous system [39]. A complete *N-Myc* knockout in the mouse reduces the size of CNS, but potential effects on later stages of CNS development could not be determined due to the limited viability beyond E11.5 [40]. In contrast to the other MYC family members, *L-Myc*-null mice are viable without obvious physiological defects or altered brain cellularity, and in the absence of qualitative changes to the distribution of *Myc* or *N-Myc* mRNA [34]. Although this might suggest that the expression of MYC and MYCN in MYCL-null cells is sufficient to functionally substitute for MYCL [41], whether MYC or MYCN compensate through an increased expression or activity is yet to be determined. Moreover, the developmental effects of the combined loss of *L-Myc* with *Myc* or *N-Myc* have not been examined [42].

**Figure 2:**
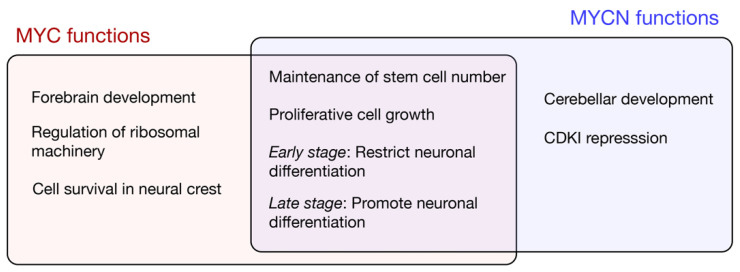
MYC and MYCN functions in the neural lineage.

Given the embryonic lethality associated with the complete loss of *Myc* or *N-Myc*, subsequent studies limited depletion to neural stem cells and intermediate neural progenitor cells using *Nestin*-Cre [43]. The general functions of MYC and MYCN uncovered using neural-specific depletion are outlined in Figure 2. A conditional *N-Myc* knockout reduces the brain size and is associated with the disorganisation of the cerebellum and cortex; the reduced proliferation and diminished cell density is associated with a decreased expression of the G1-S phase cyclin, CycD [33]. Furthermore, Cyclin Dependent Kinase Inhibitors (CDKIs) p18^INK4c^ and p27^Kip^ are upregulated following *N-Myc* knockdown, and the deletion of either CDKI partially restores the cerebellar development [44]. Given that p18^INK4c^ and p27^Kip1^ promote the differentiation of neural and oligodendrocyte precursors, respectively [45], MYCN likely functions to ensure progenitor renewal by maintaining a low expression of CDKIs during the growth phase of brain development. The mechanisms of the MYCN-mediated repression of CDKIs are unclear; while MYCN associates more weakly with the MIZ1 repressor when compared to MYC [46,47], at least in the context of prostate cancer, MYCN can also bind and recruit the PRC2 histone methylase to repress target genes [48]. The upregulation of CDKIs alone does not, however, explain the differential effects of MYCN across neural lineages. For example, *N-Myc* depletion increases the expression of the neuronal differentiation marker βTubIII/Tuj1 but does not alter the glial marker GFAP, suggesting that MYCN is required for neuronal but not glial differentiation [33]. A *Nestin*-driven *Myc* knockout also results in reduced brain growth, but with less pronounced cerebellar defects than those observed for *N-Myc* [49]. The abundant MYCN in the cerebellum likely maintains progenitor proliferation in the absence of MYC.

In accordance with region-specific MYC and MYCN functions in the brain, *Nestin*-Cre-driven double knockdown reduced forebrain and hindbrain regions at E17.5 when compared with wild type, while the midbrain was largely normal in size [41]. The combined depletion decreased neural stem cells in the forebrain, reducing proliferation and migration. The reduction was unlikely due to cell death, as apoptotic expression signatures were not observed for either the single or double loss of *Myc/N-myc* [41]. A microarray analysis of E17.5 *Myc/N-myc* double knockout brains revealed a reduced expression of genes associated with proliferative cell growth (e.g., ribosome biogenesis and cell cycle) and an increased expression of factors driving differentiation and neurogenesis, which included the upregulation of WNT signalling pathway components [41]. Nevertheless, as the expression analysis was performed on heterogeneous cell populations, direct regulatory targets of MYC in stem and progenitor cell compartments are yet to be determined.

We predict that MYC’s ability to transcriptionally upregulate the ribosomal machinery to increase the translational capacity is fundamental to the neural stem-progenitor cell transition, with a high MYC abundance promoting renewal, and decreased MYC enabling differentiation. Ectopic *Myc* overexpression in neural progenitors increases the expression of ribosomal proteins, driving progenitor proliferation and promoting brain overgrowth [50]. *Myc* expression is enriched in the E8.5 brain, but decreases at E10.5 (immediately after neural tube closure) and remains low throughout the cortical developmental stages, undergoing a 10-fold reduction in mRNA abundance without the compensatory upregulation of *N-myc* or *L-myc* [50]. *Myc* downregulation in differentiated forebrain epithelium cells correlates with the transcriptional downregulation of ribosomal proteins, translation factors and ribosomal RNA and, thus, of reduced ribosomes [50]. These changes parallel shifting metabolic requirements from glycolysis to oxidative phosphorylation, with an increased oxygenation due to the onset of placental development [51]. Thus, MYC likely promotes ribosome biogenesis in order to maintain neural stem cell renewal in the developing mammalian brain.

## 4. Neural Development in Nonmammalian Vertebrates Is Controlled by MYC

The MYC family is almost certainly required for the development of all vertebrates. In the developing chicken embryo, MYCN and MYC display complementary expression patterns in the early ectoderm; MYCN is expressed in the neural plate and neural tube [52], while MYC is expressed in neural crest cells throughout the migratory stages [53]. Although both MYCN and MYC upregulation occurs in the childhood cancer neuroblastoma, elevated MYCN is the strongest predictor of a poor prognosis [54]. Given that MYC, not MYCN, is normally expressed in neural crest cells early in chick development, MYCN amplification likely drives aberrant cell specification towards a CNS-like fate to increase tumour aggressiveness, while increased MYC in cells developmentally programmed to express MYC could explain a reduced pathogenicity [55].

The selective depletion of *MYC* on one side of chicken embryos at the gastrula stages (stage 8) reduces the pool of neural crest cells at the site of CNS origin by decreasing self-renewal and increasing cell death [53]. Interestingly, the function of MYC in the premigratory dorsal neural tube depends on interaction with the MIZ-1 transcriptional repressor, as self-renewal is not induced by the overexpression of MYC defective for MIZ-1 binding [53]. In line with this, the more potent self-renewal capacity of cultured mouse neural progenitor cells overexpressing wild type MYC is lost in MYC mutants with deficient MIZ-1 binding [56]. Furthermore, MYC overexpression promotes stemness in murine granule neuron progenitors and, consequently, inhibits neuronal differentiation, which cannot be achieved by MYCN or MYC with an impaired MIZ1 affinity [47].

Following the initiation of neurogenesis in the chick, MYC and MYCN are also detected in distinct neural cell populations: MYCN is expressed in radial glia progenitor cells within the ventricular zone, while MYC is predominantly expressed in neurons undergoing differentiation [57]. In the absence of MYCN, MYC is upregulated in the ventricular zone through a potential compensatory mechanism. The combined knockdown of *MYC* and *MYCN* in the neural tube reduces differentiated neurons, likely through a reduction of the progenitor cell pool [57]. Strikingly, overexpressed MYC in the radial glia progenitors leads to an increased number of differentiated neurons. Although maintenance of neural stem cells is also associated with inhibition of Notch signalling, the MYC and/or MYCN targets that alter Notch activity in the embryo are unknown [57]. Hence, the MYC function is required to maintain the self-renewal of neural crest cells at the early stage of embryonic development but appears to induce neural differentiation at later stages of chicken neurogenesis. In contrast with these in vivo observations, an increased proliferation in MYC-overexpressing cells isolated from embryos expanded ex vivo was associated with a failure to repress NOTCH1 expression [57], which likely required additional cues from the stem cell microenvironment. Importantly, these observations highlight the importance of understanding MYC neural functions in intact organs and tissues that more accurately recapitulate the in vivo signalling environment that instructs developmental cell fate.

Myc function has also been implicated in neural lineage specification in frog (*Xenopus*) and zebrafish neural tube and neural crest cell development. The zebrafish Myc homologue (Mych) appears to be required for neural crest cell survival as morpholino knockdown causes severe craniofacial defects [58]. Myc is highly expressed in the premigratory neural crest cells of *Xenopus*, and Myc depletion prevents neural crest formation [59]. N-Myc is expressed in the presumptive CNS of *Xenopus*, but functions of the Myc family in the developing brain require further investigation.

## 5. Nonvertebrate Models Reveal Myc Roles in Neural Stem Cells

**Figure 3:**
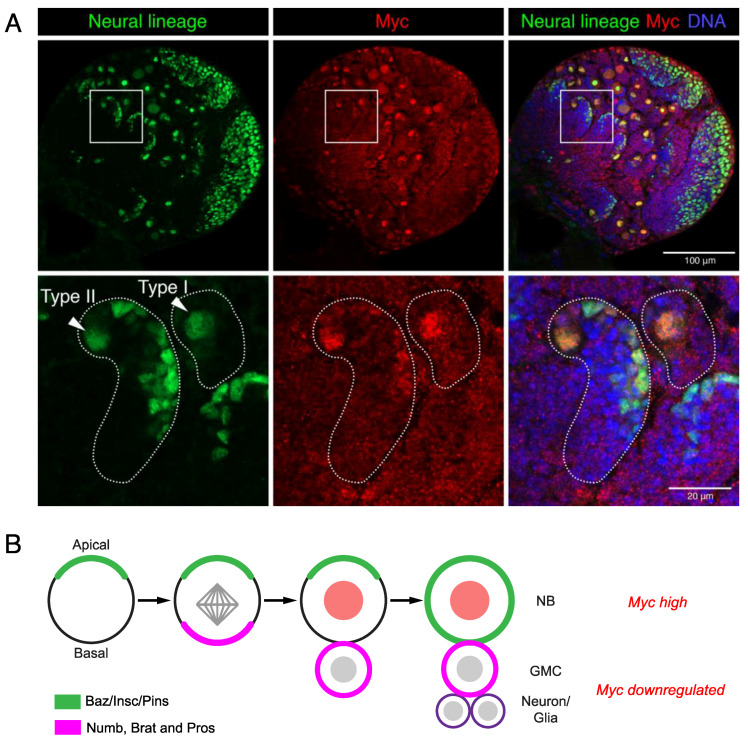
Myc promotes *Drosophila* neural stem cell renewal. (**A**) Myc is high in neural stem cells in the larval brain (neural lineage marked with Deadpan, green). Type I neuroblasts (NB) directly produce ganglion mother cell (GMC) progenitors, while Type II produce GMCs via a transit-amplifying intermediate progenitor lineage. (**B**) Neuroblasts divide asymmetrically in order to self-renew and generate progenitors. Apical and basal factors are asymmetrically distributed during mitosis in order to specify the stem and progenitor cell fate of the daughters.

Neural stem cell determinants are conserved between vertebrates and invertebrates; thus, *Drosophila* models have provided significant insights into neural stem cell fate control. The *Drosophila* neural stem cells or neuroblasts (NBs) undergo asymmetric cell division for self-renewal in order to maintain the stem cell population and generate intermediate progenitors that differentiate to give the neuronal and glial populations that comprise the developing brain (reviewed in [60]). The single Myc family protein is highly expressed in neuroblasts and is decreased in progenitors [61] (Figure 3A).

Myc levels are controlled by a variety of mechanisms to ensure tight control of the asymmetric neuroblast division in the *Drosophila* larval brain. Brain tumour (brat) controls *Myc* post-transcriptionally by translational repression in the progenitors (called ganglion mother cells/GMCs in *Drosophila*) [61]. Brat protein establishes neuroblast polarity, which is essential for the correct mitotic spindle alignment and distribution of cell fate determinants between the stem and daughter cells (reviewed in [60,62,63,64]). Basal localisation in neuroblasts and the subsequent distribution of stem cell fate determinants, such as Prospero and Numb, into progenitor cells drives the differentiation of GMCs [65,66,67] (Figure 3B). Perturbations to the asymmetric cell division drive stem cell renewal over differentiation, resulting in “brain tumour” phenotypes; for example, brat loss-of-function drives the overproliferation of neuroblasts at the expense of progenitor differentiation [68]. The failure of *brat* mutant progenitors to downregulate Myc protein likely promotes proliferation at the expense of differentiation [61]. The mammalian ortholog of brat, TRIM3, has similarly been shown to reduce *MYC* and promote differentiation in human glioma cell lines, while a reduced TRIM3 expression has been reported in glioblastoma and is associated with poor survival [69].

More recent studies implicate the regulation of *Myc* mRNA stability by the RNA-binding proteins Imp (conserved as mammalian Insulin Growth Factor (IGF) 2 mRNA-binding protein/IGF2BP2) and Imp antagonist Syp (Syncrip in mammals), as key determinants of neuroblast renewal [70]. Genome-wide single molecule fluorescent in situ hybridisation identified *Myc* as an Imp RNA binding target. Moreover, *Myc* mRNA stability was increased following Imp overexpression. On the other hand, the Imp antagonist Syp reduced *Myc* mRNA stability indirectly by negatively regulating Imp [70]. Thus, knockdown of Imp in the neural lineage reduced the cell size and proliferation of type I neuroblasts, while *Syp* knockdown increased proliferative growth [70]. Mammalian IGF2BP proteins also appear to play a conserved role in stabilising *MYC* transcripts [71] and are overexpressed in glioblastoma [72].

The Notch pathway is evolutionarily conserved and widely regulates cell fate specification during both development and homeostasis in the adult [73,74]. Notch signalling was first characterised in the fly and named from the notching in the adult wings in heterozygous loss-of-function mutants. Notch activation, via overexpression of the intracellular domain, increases the stem cell number in the larval brain [75]. ChIP assays for the key transcription factor downstream of Notch, suppressor of Hairless (Su(H)), revealed an enrichment on *Myc* in the larval brain [76]. Moreover, *Myc* promoter activity was increased in mutants for the Notch pathway inhibitor Ada [76]. Together, these observations suggest that Notch transcriptional effectors activate *Myc* in *Drosophila* neuroblasts, providing a mechanistic link between Notch signalling, Myc activation and stem cell renewal.

In line with mammalian MYC’s capacity to drive the activation of the protein biosynthesis machinery in the developing forebrain [50], *Drosophila* Myc directly activates the transcription of the eukaryotic translation initiation factor 4E (eIF4E) in neuroblasts [76]. Although the interaction between eIF4e and ribosomes is largely regulated by post-translational changes (e.g., phosphorylation by TOR pathway kinases [77,78]), the overexpression of Rheb, an upstream component of the TOR pathway, is insufficient to suppress the stem cell depletion associated with Notch inhibition in neuroblasts. On the other hand, Myc overexpression restores stem cell loss, implicating Myc as a key downstream target in the Notch-dependent control of neuroblast identity [76].

Myc further maintains neuroblast renewal through interaction with the Tip60 transcriptional activator complex [79], which also regulates Myc-dependent transcription in mouse embryonic stem cells [80]. In *Drosophila*, an in vitro binding assay in S2 cells revealed a direct interaction between Myc and Tip60 complex subunits, Pint and Rept [81], while a physical interaction with Bap55 was observed in embryos with mass spectrometry [79]. Moreover, the neural lineage-specific knockdown of either *Myc* or the Tip60 subunit *Dom* disrupts stem cell polarity to prematurely drive Prospero-dependent differentiation and, ultimately, neuroblast depletion [79]. Common binding targets of Myc and Dom, based on ChIP enrichment in the larval brain, include spindle and centrosome positioning genes such as aPKC [79]. Moreover, aPKC appears to be a key target as the mislocalisation of polarity determinants (Mira and Baz) in *Myc* or *Dom* knockdown neuroblasts are restored by aPKC overexpression [79]. However, aPKC overexpression does not completely restore the spindle organisation associated with the knockdown of *Myc* or *Dom*, which suggests that additional target genes of the Myc-Tip60 transcriptional network are required for a proper asymmetric division. Thus, future studies are required to determine the full complement of Myc binding targets specifically in the neural stem cell compartment and the significance to Myc-dependent neuroblast renewal.

## 6. Brain Tumours Are Driven by Cancer Stem Cells

Although cancer stem cells were characterised in brain tumours almost two decades ago [82,83], little progress has been made towards the therapeutic targeting of glioma stem cells to improve survival for this group of cancer patients [84]. Currently, brain cancer is the most common paediatric malignancy, while the median survival time for glioblastoma, the most frequently occurring adult brain cancer, is only 14 months [85]. The inherent heterogeneity and complexity of these invasive stem cell-driven cancers have severely impeded therapeutic advancement. Emerging evidence, supported by single-cell RNA sequencing, suggests that the dedifferentiated glioma stem cell state characteristic of brain tumours arises from the disruption of developmental networks essential for controlling neural stem cell fate and brain formation [86,87]. As in neural stem cells, MYC likely promotes glioma by amplifying transcriptional programs promoting renewal and hampering differentiation. Thus, studies into MYC’s developmental roles are essential to provide insights into mechanisms of dysregulation during tumorigenesis.

## 7. Conclusions and Future Perspectives

Collectively, model organism studies have provided valuable insights into MYC family functions in development. Across all metazoans studied to date, MYC is universally required to maintain the growth and proliferation of neural stem cells. However, MYC is additionally seen within the differentiating murine neural progenitors, indicative of the importance of a tightly regulated expression in directing cell fate.

Despite the molecular characterisation of brain tumours by The Cancer Genome Atlas [88,89], novel therapies have failed to make it into the clinic, with an inability to pass phase III clinical trials [90,91]. Brain tumours resist conventional and novel treatments, at least in part, due to the combination of unique cell-intrinsic and microenvironmental properties of neural tissues. In 2019, a Cancer Research UK gathering of brain cancer clinicians and researchers identified seven major challenges for curing brain cancer [92], with a major impediment to progress in brain cancer treatment being our poor understanding of the tumour microenvironment. The prediction is that cell-cell communication between glioma stem cells and the surrounding microenvironment will be integral to tumour initiation, progression and response to therapies. Studies into cell-cell communication in glioma are largely hindered by normal brain complexity and the inability to conduct in vivo functional studies. Thus, the molecular basis underlying the behaviour of heterogeneous glioma cells is limited to knowledge from ex vivo tumour studies, particularly glioma monocultures and patient-derived xenografts in immunocompromised mouse hosts. Drugs developed using these approaches will target the tumour but are likely to fail on a clinical level as these models do not recapitulate in vivo tumour behaviour.

Neural stem cell fate is regulated externally by neurotransmitters, cerebrospinal fluid and surrounding vasculature [93]; glioma stem cells will similarly be maintained through complex exogenous signals in the tumour microenvironment. To date, very few studies have investigated the interplay between the neural microenvironment and MYC regulation in the context of glioma. One report suggested that extracellular vesicles secreted by glioblastoma tumour cells were sufficient to increase *MYC* and reduce *TP53* mRNA in wild-type astrocytes, although the mechanisms were unclear [94]. An increased cell migration was observed in primary astrocytes incubated with glioblastoma-derived extracellular vesicles, and a proteomic analysis revealed the upregulation of eukaryotic translation initiation eIF2 and mTOR pathway components, indicating that communication with the surrounding tumour microenvironment supported tumour expansion [94].

In vivo developmental genetic models will continue to be a major tool for understanding glioma biology. Moreover, as molecular mechanisms controlling neural stem cell fate are conserved between mammals and *Drosophila*, the sophisticated lineage-specific genetic manipulation in the latter will enable the dissection of the molecular mechanisms underlying Myc’s capacity to maintain stemness [95]. As in mammals, glial cells in *Drosophila* cover the surface of the CNS and provide the niche required for neural stem cell maintenance and differentiation [96,97]. Moreover, the capacity to specifically manipulate gene expression in either stem cells themselves or the surrounding stem cell microenvironment (or niche) will enable the dissection of the relative contribution of nonautonomous signalling from the microenvironment to neural stem cell fate. More recently, 3D neural tissue culture techniques have been developed for the generation of human brain organoids (minibrains), which have the potential to shed light on tumour-microenvironment interactions [98,99]. Together, the *Drosophila* and human models will provide crucial insights into MYC functions in neural stem cell fate control and dysregulation in glioma, in the context of both normal and tumour microenvironments.

## Abbreviations

bHLH	basic helix-loop-helix
CDKI	Cyclin Dependent Kinase Inhibitor
ChIP	Chromatin Immunoprecipitation
CNS	central nervous system
E	embryonic day
GMC	ganglion mother cell
NB	neuroblast
PNS	peripheral nervous system
